# The Occurrence of *Colletotrichum karstii* and *C. fructicola* Causes Anthracnose on Endangered Ethnic Vegetable *Yunnanopilia longistaminea* in Yunnan, China

**DOI:** 10.3390/jof11100748

**Published:** 2025-10-19

**Authors:** Jian-Wei Guo, Rong-Chuan Tian, Chun-Lian Yang, Lizhi Jia, Su-Yue Zhou, Min Yang, Lifang Li, Penghua Gao, Lei Yu, Murad Muhammad, Ming-Liang Ding, Shi-Kang Shen

**Affiliations:** 1 College of Agronomy and Life Sciences, Yunnan Urban Agricultural Engineering and Technological Research Center, Kunming University, Kunming 650214, China; trww2017@163.com (R.-C.T.); 15894424392@163.com (C.-L.Y.); palpitate_ho@163.com (L.J.); suyuezhou@outlook.com (S.-Y.Z.); yangmin7799@163.com (M.Y.); csqsmile@126.com (L.L.); gaopenghua8878@dingtalk.com (P.G.); yulei0425@163.com (L.Y.); 2 State Key Laboratory of Desert and Oasis Ecology, Key Laboratory of Ecological Safety and Sustainable Development in Arid Lands, Xinjiang Institute of Ecology and Geography, Chinese Academy of Sciences, Urumqi 830011, China; muradbotany1@uop.edu.pk; 3 University of Chinese Academy of Sciences, Beijing 100049, China; 4 Food Crops Research Institute, Yunnan Academy of Agriculture Sciences, Kunming 650200, China; dml@yaas.org.cn; 5 State Key Laboratory for Vegetation Structure, Function and Construction (VegLab), Ministry of Education Key Laboratory for Transboundary Ecosecurity of Southwest China, Yunnan Key Laboratory of Plant Reproductive Adaptation and Evolutionary Ecology, Institute of Biodiversity, School of Ecology and Environmental Science, Yunnan University, Kunming 650504, China

**Keywords:** *Yunnanopilia longistaminea*, *Colletotrichum karstii*, *Colletotrichum fructicola*, ethnic vegetable

## Abstract

The study investigates the morphological and phylogenetic characteristics of *Colletotrichum* species linked to anthracnose on *Yunnanopilia longistaminea* (*Opiliaceae*) in Yunnan, China. From July 2023 to July 2024, foliar anthracnose on *Y. longistaminea* 20-year-old plants with an incidence rate of 16% and two-year-old seedlings with an incidence rate of >90% were investigated in Yunnan, China. Based on morphological features and molecular approaches, four isolates of *Colletotrichum* were identified as *C. karstii* and *C. fructicola*. Two species were verified to induce foliar anthracnose by validating Koch’s postulates. This is the first report of *C. karstii* and *C. fructicola* inducing foliar anthracnose on *Y. longistaminea* in Yunnan, China, and globally. These findings enhance our understanding of the fungal pathogens affecting *Y. longistaminea* leaves and provides a theoretical basis of conservation and disease management in the study area. Further research is needed to explore these species’ ecological impacts and potential control measures in agricultural practices and wild resources protection.

## 1. Introduction

There are 13,253 species of seed plants belonging to 2140 genera and 225 families in Yunnan Province accounting for 4.1% of the country’s total area [1], making it the province with the highest number of plant species in China [2], and one of the most significant biodiversity hotspots in the world [3,4]. However, habitat loss or fragmentation, resource overuse, invasion of alien species, environmental pollution, human-induced climate change, and barriers to biological reproduction have negatively affected biodiversity [5].

The family *Opiliaceae* consists of about 38 species and 12 genera, among which *Cansjera* Jussieu, *Lepionurus* Blume, *Opilia* Roxburgh, *Urobotrya* Stapf and *Yunnanopilia* Wu & Li are found in Yunnan Province, China [6]. At the same time, *Champereia* Griffith is only distributed in Taiwan Province, China [6]. *Y. longistaminea* (W.Z. Li) C.Y. Wu & D.Z. Li (syn: *Melientha longistaminea* W.Z. Li) is an evergreen tree that is mainly distributed in the deciduous monsoon forest of the Red River area in the Yunnan and Guangxi Provinces of China, North Vietnam and Laos [7,8]. Its tender leaves and stems are rich in crude protein, total amino acids, vitamin C, potassium and crude fibre [9,10]. Therefore, it is significant for the local ethnic minorities and has been cultivated since 2001 [11]. Nowadays, it is mainly cultivated in the Red River area including Yuanjiang County, Mojiang County, Shiping County, Xinping County and Shuangbai County, in Yunnan Province, China. According to the investigation, the price of tender leaves and stems is about 120–200 RMB/kg, and the income is more than 450,000 RMB/hm^2^. Therefore, the domestic cultivation of *Y. longistaminea* increases the income of local ethnic minorities and reduce wild resource overuse.

In China, previous studies have focused on phylogenetic placement, morphological characteristics, karyotype, distribution, nutritional composition, tissue culture, and domestication cultivation [6,8,9,10,11,12,13,14,15]. However, the disease and pest were rarely reported on the family *Opiliaceae* especially the species *Y. longistaminea*. Up to now, only *Umbaspis regularis* was reported as the pest of *Agonandra brasiliensis* Miers ex Benth. & Hook.f. (Opiliaceae) in Brazil [16].

The genus *Colletotrichum,* consisting of 340 species with about 1400 known host plant species, is recognized as epiphytes, endophytes or saprobe [17], with notable importance as one of the top 10 fungal pathogens [18]. Among them, *C. siamense*, *C. fructicola*, *C. karstii*, *C. fioriniae* and *C. gloeosporioides* are the top 5 species described as plant pathogens [19]. They usually cause anthracnose, resulting in spots, blight or rot on leaves, flowers, fruits, stems, and postharvest vegetables [20]. For example, *C. musae* can lead to 30–40% banana losses of marketable fruit [19,20].

Recently, studies have showed that many crops cultivated worldwide are usually infected by multiple pathogens of the same genus [18,21,22], and the dominant species vary according to geographical distribution or host varieties. For instance, among 10 species of *Colletotrichum* isolated from maize anthracnose in Sichuan Province, *C. cliviicola*, *C. fructicola*, *C. siamense*, *C. karstii*, and *C. truncatum* were confirmed as pathogens by pathogenicity tests. The dominant pathogens in Chengdu City were *C. cliviicola*, *C. fructicola* and *C. karstii*, whereas in Meishan City, the dominant species was *C. karstii* [23]. Additionally, three novel species and five known species of *Colletotrichum* were isolated from walnut anthracnose in Shanxi and Sichuan Province, China [24]. In Western Australia, *C. gloeosporioides*, *C. karstii* and *C. novae-zelandiae* were identified as the cause of twig dieback of citrus, with *C. gloeosporioides* being recognized as the most aggressive and dominant species [25].

In July 2023 to July 2024, anthracnose was frequently discovered on plants of *Y. longistaminea* in Honghe Prefecture and Kunming City, Yunnan Province, China. Thus, this article aimed to make clear how many species can cause anthracnose on plants of *Y. longistaminea*, and which is the dominant pathogen by identifying the pathogen based on morphological characteristics and multiple loci phylogenetic analysis.

## 2. Materials and Methods

### 2.1. Sampling Sites, Diseased Sample Collection and Pathogen Isolation

A 53 ha plantation of *Y. longistaminea* is located in a hillside in Xincheng Town, Shiping County, Honghe Prefecture, Yunnan Province, China (GPS Coordinates: 23.780421° N, 102.477260° E, altitude 1393.5 m). Those plants were 20 years old with heights ranging from 1.5 to 3.0 m. A greenhouse with about 200 two-year-old seedlings of *Y. longistaminea* is located in Kunming Botanical Garden, Chinese Academy of Sciences, Kunming City, Yunnan Province, China (GPS coordinates: 25.137383° N, 102.743551° E, altitude 1587.1 m).

In July 2023 and July 2024, the anthracnose incidence rate (IR) of *Y. longistaminea* were investigated using the following formula: IR = (disease plants/total number of plants investigated) × 100%. Diseased leaves were randomly collected and transferred to the phytopathology laboratory of Kunming University.

The pathogens were isolated according to the methods described by Guo et al. [26] with some modification as follows: Five diseased leaves were washed by running tap water, then surface sterilized by 75% ethanol for 30 s, 2.5% sodium hypochlorite for 1 min, rinsed five times with sterilized distilled water. Subsequently, tissue pieces (5 × 5 mm^2^) were cut from the necrotic spots and dried by sterilized filter paper, and finally transferred onto rose bengal agar (RBA; 5.00 g peptone, 10.00 g glucose, 1.00 g potassium dihydrogen phosphate, 0.50 g magnesium sulphate, 0.03 g rose bengal sodium salt, 0.10 g chloromycetin, 15.00 g agar per L) in Petri dishes at 25 °C for three days. The germinated mycelial tips were ticked and transferred to fresh potato dextrose agar (PDA; 200.00 g potatoes, 20.00 g glucose, 0.10 g chloromycetin, 15.00 g agar/L) plates to obtain pure culture. All the pure cultures were preserved at the Yunnan Urban Agricultural Engineering and Technological Research Center, Kunming University.

### 2.2. Pathogenicity Test

All the isolates were grown on PDA at room temperature (16–25 °C) for fourteen days. Conidia were harvested by pouring 10 mL sterilized 0.05% Tween-80 into the plates, and then, conidial suspension was adjusted to 10^6^ conidia/mL using a haemocytometer. Thirty needle-wounded healthy leaves of ten two-year-old seedlings were sprayed 2–3 cm above the leaves with 1.5 mL conidial suspension of each isolate. Other thirty wounded healthy leaves were sprayed with 1.5 mL sterilized water containing 0.05% Tween-80 as control [27]. All the leaves were covered by sterilized absorbent cotton. Then, all the seedlings were covered by plastic bags to remain in the humidity for 10 days and placed into a greenhouse under 20–30 °C. Finally, the artificial inoculated leaves were used to re-isolate the pathogen according to the method described in Section 2.1.

### 2.3. Morphological Identification

All the pathogenic isolates were inoculated on PDA at 28 °C in the dark. Each pathogenic strain’s colony characteristics, conidial shape, and size were measured and photographed under a light OlympusBX53 microscope (Olympus Optical Co., Ltd., Tokyo, Japan) [28]. Twenty or thirty conidia of each isolate were randomly selected to measure the morphology [23,28].

### 2.4. DNA Extraction, Sequencing, and Analysis

The pathogenic isolates were inoculated on PDA at 28 °C in the dark for two weeks, and then mycelia were collected from the colony surfaces. Genomic DNA extraction was performed using the Solarbio Fungi Genomic DNA Extraction Kit (Beijing Solarbio Science & Technology Co., Ltd., Beijing, China) following the manufacturer’s protocol. This procedure ensures efficient and reliable extraction of genomic DNA suitable for subsequent analyses. The internal transcribed spacer of the rDNA (ITS), glyceraldehyde-3-phosphate dehydrogenase (GAPDH), actin (ACT), and β-tubulin 2 (TUB2) were amplified and sequenced using the primer pairs ITS-1/ITS-4 for ITS [29], GDF/GDR for GAPDH [30], ACT-521F/ACT-783R for ACT [31], and T1/Bt2b for TUB2 [32], respectively. PCR amplification system was carried out according to the reaction described by Santos et al. [27], and the PCR parameters presented by Truong et al. were followed [33]. The amplified PCR products were used to sequence by Tsingke Biotechnology Co., Ltd., Beijing, China, a commercial sequencing service.

The sequences were analyzed by BLAST searches (blastn) (https://blast.ncbi.nlm.nih.gov/Blast.cgi?PROGRAM=blastn&PAGE_TYPE=BlastSearch&LINK_LOC=blasthome, accessed on 16 March 2025) of the GenBank database and submitted to NCBI GeneBank (https://www.ncbi.nlm.nih.gov/, accessed on 16 March 2025). Then, the sequences of 13 *Colletotrichum* ex-type were downloaded from GenBank. All the sequences of the four gene fragments (ITS-GAPDH-ACT-TUB2) of 13 *Colletotrichum* ex-type and 3 pathogenic isolates in this study were firstly aligned with ClustalX [34], respectively. Then, they were manually concatenated according to the ITS, GAPDH, ACT, and TUB2 order. Finally, a neighbour-joining (NJ) tree based on the four gene fragments was constructed with the Maximum Composite Likelihood method (ML) using Molecular Evolutionary Genetics Analysis (MEGA) (version 7.0) [35]. The bootstrap values with 1000 replicates were carried out to test branch support.

## 3. Results

### 3.1. Symptomatology and Fungal Isolation

In July 2023, anthracnose symptoms were discovered on 20-year-old plants of *Y. longistaminea* in a hillside in Xincheng Town, Shiping County, Honghe Prefecture, Yunnan Province, China. The plum blossom method investigated the symptom and incidence rate at the four corners and centre [36]. A total of 16% of the plants surveyed on 250 plants displayed anthracnose symptoms on their lower mature leaves. In July 2024, >90% of leaves surveyed on about 200 two-year-old seedlings in a greenhouse in the Kunming Botanical Garden (Chinese Academy of Sciences, Kunming City, Yunnan) showed symptoms of anthracnose, with around 50% of the affected leaves subsequently abscising.

**Figure 1:**
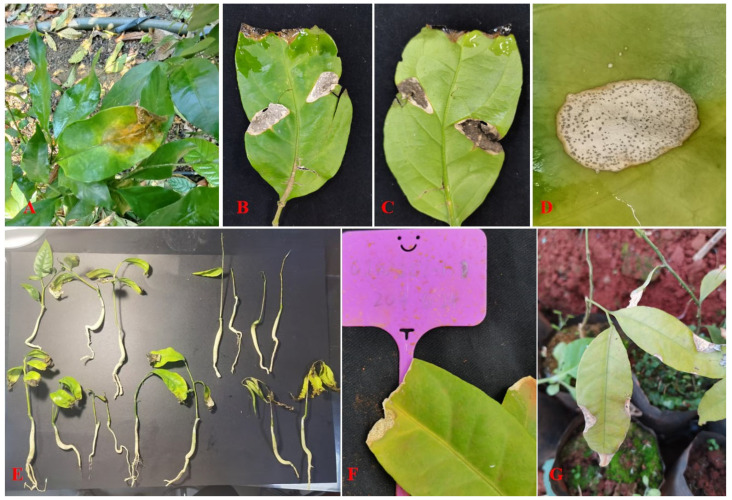
Naturally infected (**A**–**E**) and artificially inoculated (**F**,**G**) symptoms of anthracnose on *Yunnanopilia longistaminea* in Shiping County, Yunnan Province, China. (**A**) Naturally symptom of anthranose on *Y. longistaminea* 20-year-old plant; (**B**) the top view of natural symptom of anthracnose; (**C**) the dorsal view of natural symptom of anthracnose; (**D**) the necrotic spot of anthracnose; (**E**) naturally diseased two-year-old seedlings of *Y. longistaminea* in Kunming City, Yunnan Province, China; (**F**) the anthracnose symptom of *Y. longistaminea* twenty days of inoculation with conidial suspension of GJW200-1; and (**G**) the anthracnose symptom of *Y. longistaminea* twenty days of inoculation with conidial suspension of GJW202-2. All the treatments were maintained at 20–30 °C at a relative humidity of 45 to 100% in a greenhouse.

Initially, red-brown or light brown needle-point spots appeared on both sides or tips of the leaves, gradually expanded, and some neighbour spots coalesced into one more prominent spot. The boundary between the necrotic and surrounding parts was obvious. The border of the necrotic spot was brown with an indistinct yellow halo. The necrotic spot turned grey-white and formed black concentric rings with a target-like appearance consisting of conidia and conidiophores (Figure 1A–E). Finally, the diseased leaves developed holes, or the diseased spots turned black and covered some grey or green mould when the air humidity was saturated. Then, entire leaves curled and fell off.

Following tissue separation and single-spore isolation, four isolates, GJW200-1, GJW200-2, GJW202-1 and GJW202-2, were isolated and clustered into two groups based on colony characteristics.

### 3.2. Pathogenicity Test

Through needle-wound inoculation, four isolates of *Colletotrichum* could cause similar symptoms to those observed in the field and the greenhouse. After two weeks of inoculation with the four isolates, namely GJW200-1, GJW200-2, GJW202-1 and GJW202-2, at 20–30 °C and a relative humidity of 45 to 100%, all the artificially infected leaves began to appear some water-soaked dark-green or light-brown, needle-point spots on both sides or tips, gradually enlarged and turned grey-white with dark-brown border and yellow halo, then produced some black concentric rings (conidiomata) with a target-like on the necrotic spots (Figure 1F,G). Finally, some diseased leaves curve, turn yellow-brown, develop holes, or fall off. All *Colletotrichum* isolates were re-isolated from the inoculated leaves, while no *Colletotrichum* isolate was recovered from control leaves, fulfilling Koch’s postulates.

### 3.3. Morphological and Molecular Identification of the Pathogen

**Figure 2:**
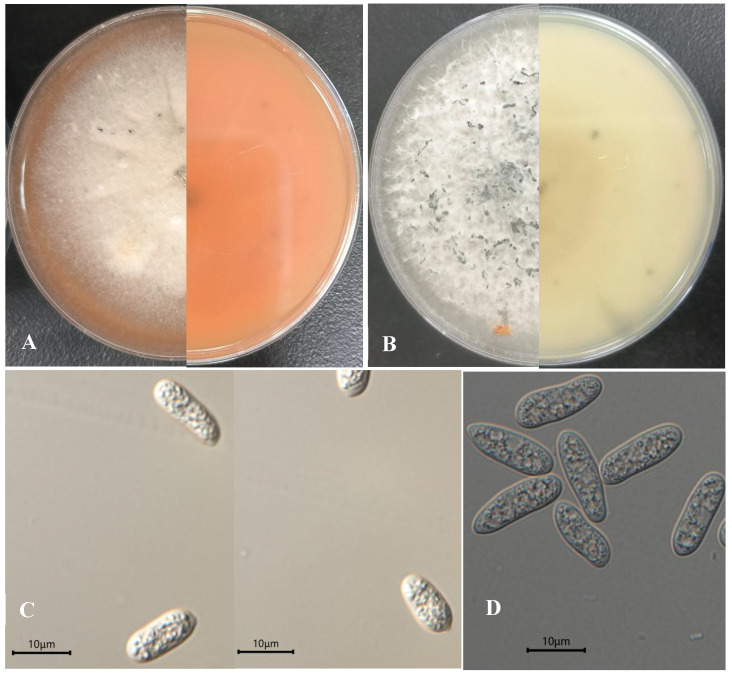
(**A**). The colony front and reverse view of *Colletotrichum karstii* GJW200-1 after 9 days of inoculation on PDA at 28 °C in the dark; (**B**) the colony front and reverse view of *C. fructicola* GJW202-2 after 9 days of inoculation on PDA at 28 °C in the dark; (**C**) the conidia of *C. karstii* GJW200-1; (**D**) the conidia of *C. fructicola* GJW202-2. Bars = 10 µm.

The colony diameter of all the strains growing on PDA at 28 °C after inoculation 9 days was 90 mm. Colonies of GJW200-1 and GJW200-2 on PDA at 28 °C after 9 days were white and produced sparsely aerial mycelia at the centre and scatter of tufts. On the reverse side, the colony is orange-red. Conidia were hyaline, smooth-walled, aseptate, straight, short cylindrical with circular ends and 10.5–16.7 × 3.6–6.3 µm (mean 13.8 × 5.5 µm; *n* = 30) in size (see Figure 2A,C). These characteristics were similar to *Colletotrichum karstii* Y.L. Yang, Zuo Y. Liu, K.D. Hyde & L. Cai [12–19.5 × (5–) 6–7.5 µm (mean 15.4 ± 1.3 × 6.5 ± 0.5)] [37], whereas colonies of GJW202-1 and GJW202-2 on PDA at 28 °C after 9 days were white, dense, cottony, and turned green after 7–9 days. On the reverse side, the colony was still white to grey-white. Conidia were hyaline, smooth, aseptate and cylindrical with rounded ends and 14.4–18.4 × 4.9–6.5 µm (mean 16.4 × 5.7 µm; *n* = 20) in size (See Figure 2B,D). Those characteristics were morphologically consistent with *Colletotrichum fructicola* Prihastuti, L. Cai & K.D. Hyde [9.8–17.7 × 4.5–5.9 µm (mean 12.5 ± 0.7 × 5.1 ± 0.4] [38].

**Figure 3:**
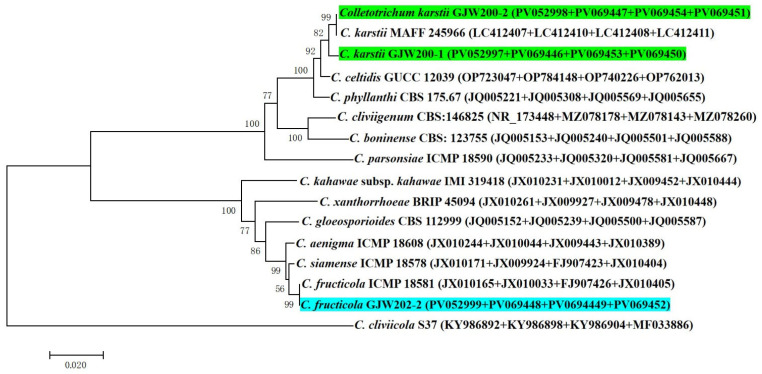
N-J phylogenetic trees of *Colletotrichum karstii* GJW200-1, GJW200-2 and *C. fructicola* GJW202-2 based on ITS-GAPDH-ACT-TUB2 sequences. The isolates from this study are indicated in green or blue at the bottom. The scale bar shows the number of expected changes per site.

Three of the four pathogenic isolates were used for multiple loci phylogenetic analysis. The aligned concatenated sequences of 13 strains of *Colletotrichum* ex-type and three isolates from *Y. longistaminea* in the present study contained 1369 characters, including gaps (ITS: 1–517; GAPDH: 518–628; ACT: 629–813; TUB2: 814–1369). The phylogenetic trees showed GJW200-1 and GJW200-2 clustered in a clade with *C. karstii* MAFF 245966, and GJW202-2 clustered together with *C. fructicola* ICMP 18581 (see Figure 3).

## 4. Discussion

The genus *Colletotrichum* has a more than 100-year history as a model pathogen for pathogenicity differentiation [39], functional genes such as the cyclic adenosine monophosphate (cAMP), MAPK, RAS/small G-protein and calcium-mediated signalling pathways, conidial germination, appressorium development [40,41,42,43,44], systemic acquired/induced resistance (SAR) [45] and phytoalexins [46]. Thus, it is recognized as one of the top 10 fungal pathogens [18].

Accuracy in species identification based on pure culture is the indispensable foundation of agriculture, including accurate control of plant disease, breeding, conservation biology, ecosystem management, medicine, and so on [21,47,48,49]. GJW200-1 and GJW200-2 were clustered in a clade with *C. karstii* and neighbour to *C. celtidis* and *C. phyllanthi*; in contrast with the size of *C. karstii* (12.6–20.8 × 5.5–8.9) µm, that of *C. celtidis* was 10.3–18.4 × 5.2–8.0 µm and that of *C. phyllanthi* was 10.4–15.8 × 5.7–8.8 µm [50], the conidial size of GJW200-1 and GJW200-2 [10.5–16.7 × 3.6–6.3 µm (mean 13.8 × 5.5 µm)] was also similar to *C. karstii*. Therefore, GJW200-1 and GJW200-2 were identified as *C. karstii* based on both morphological and phylogenetic analysis. *C. karstii* could cause anthracnose of *Synsepalum dulcificum* (miracle fruit) in Japan [33], walnut in China [24], citrus in Western Australia [25] and Chayote in Brazil [51]. Whereas, GJW202-2 was clustered in a clade with *C. fructicola* and neighbour to *C. siamense and C. aenigma*, additional the conidial size of GJW202-2 [14.4–18.4 × 4.9–6.5 µm (mean 16.4 × 5.7 µm)] was also similar to that of *C. fructicola* (15.9 × 5.2 µm) comparison with that of *C. siamense* (15.6 × 5.1 µm) and *C. aenigma* (16.2 × 5.0 µm) [52]. Therefore, GJW202-2 was identified as *C. fructicola* combined with morphological and molecular characteristics. In China, *C. fructicola* has been discovered as the anthracnose agent of maize [23], and pepper [53]. Although the microbiome provides a quick and novel path to identify known or new pathogens [54], Koch’s postulates are still considered the gold standard for establishing causality between microbes and diseases [55]. Thus, this is the first report of *C. karstii* and *C. fructicola* causing foliar anthracnose of *Y. longistaminea* throughout Koch’s postulates in Yunnan, China, and worldwide. It is consistent with the conclusion that every plant cultivated worldwide is usually infected by multiple pathogens of *Colletotrichum* [18].

The resistance of plant tissue to pathogens is related to age [56], and most plants have weaker disease resistance in early development than in late development [57], which might be demonstrate that why higher incidence on two-year-old seedlings (>90%) compared to 20-year-old trees (16%). Additionally, two-year-old seedlings are cultivated in a green house with higher humidity and temperature while 20-year-old trees are cultivated in the field, which stated temperature and humidity influence the incidence and disease severity of anthracnose on *Y. longistaminea.* A previous study showed there was a delay of incubation and latent period when plants were left in a non-humid open environment than when exposed to wetness durations and higher temperature after inoculation [58]. Therefore, it is necessary to further investigate the effect of host development stage, temperature and humidity on pathogenicity of *C. karstii* and *C. fructicola* on *Y. longistaminea*.

The investigation of disease incidence indicated the lower older leaves on *Y. longistaminea* is susceptible to anthracnose; moreover, 97% carbendazim WP exhibited the best inhibitory effect on anthracnose caused by *C. fructicola*, with an EC_50_ (concentration for 50% of maximal effect) value of 0.0242 µg/mL [59]. Thus, the removal of the underlying old leaves and the use of 97% carbendazim WP will be beneficial to control the anthracnose of *Y. longistaminea* cultivated in the field.

In Yunnan Province, China, *Bauhinia* × *blakeana* Dunn, *Metapanax davidii* (Franch.) J. Wen ex Frodin, *Eleutherococcus senticosus* (Rupr. & Maxim.) Maxim., *Dregea volubilis* (L. f.) Benth. ex Hook. f., *Crateva unilocularis* Buchanan-Hamilton and *Eryngium foetidum* L. are being domesticated as ethnic vegetables. It is helpful to reduce wild resource overuse; however, their pathogen identification and fungicide screening are rarely carried out.

## 5. Conclusions

This research found that *C. karstii* and *C. fructicola* cause foliar anthracnose of *Y. longistaminea* in Yunnan, China, and worldwide. The incidence rates of 16% on 20-year-old plants in the field and more than 90% on 2-year-old seedlings in a greenhouse might result in a significant loss in the region.

## Data Availability

The sequences of GJW200-1, GJW200-2 and GJW202-2 (PV052997~PV052999 for ITS, PV069446~PV069448 for GAPDH, PV069450~PV069452 for TUB2), and the ACT gene of GJW200-1 (PV069453), GJW200-2 (PV069454) and GJW202-2 (PV069449) have been submitted to NCBI and opened to the public.
